# Proposed Emergency Hospital for Ilford

**Published:** 1904-12-17

**Authors:** 


### PROPOSED EMERGENCY HOSPITAL FOR ILFORD.

A SPECIAL meeting of the Hospital Committee of the Ilford Medical Society was held in the Council Chamber at Ilford Town Hall on Tuesday afternoon, Dr. A. B. Cruickshank presided. The following address by Dr. Richard Greene, F.R.C.P., was given.

Modern hospitals are invariably built on some modification of the pavilion system, and it is therefore not necessary to refer to any other method of construction.

This pavilion system, which is really a collection of hospital units, has only one drawback, and that is the amount of land required for its proper construction. This amount of land cannot be accurately stated as so much per bed, or per 50 beds, because, other things being equal, the larger the hospital the less amount of land it will, relatively, require. It will also be modified by the height of the pavilions, and by the space which it is possible to obtain; but assuming that the blocks be three storeys high, this would give a total height, from ground-level to ridge, of about 65 feet, and the pavilions must never be placed nearer each other than this height, which is, in fact, the barest minimum, and I look upon it as quite insufficient. St. Thomas's Hospital, in London, has 130 feet between pavilions. At the new Derby hospital it is 100 feet; at

Bedford 105 feet; and at the new infirmary for Northampton, which has lately been finished, in conjunction with Mr. Dorman, it is 155 feet.

Whether the pavilions be three storeys or two storeys or only one storey in height does not make very much difference in the distance which it is desirable to obtain between the pavilions. I am of opinion that the best height is one storey only. By adopting this plan we are at one stroke freed from the necessity of providing staircases, lifts, and fire-escapes, and, furthermore, the whole work of the institution can be more easily carried on—meals are more quickly served, supervision is easier, there is no ward overhead to interfere by its noise or otherwise with the one underneath, and repairs are more easily carried out. Of course, the cost per bed is somewhat greater, but it is not nearly so much greater as would be thought at first sight—that is, apart from the extra land which the longer extension of the blocks renders necessary. The administrative portions of the building are better of two storeys or even of three.

Now as to the quantity of land required. The Derby infirmary, which I have already mentioned, has 196 beds and 13½ acres of land; Bedford has 100 beds and 10 acres; Northampton has 160 beds and 15 acres. I need scarcely say that these larger sites are not required merely for the building to stand on, but some of the land should be adapted for the recreation of the patients, and every hospital should have around it a belt of shrubs, to prevent its being overlooked, but such trees should never be high enough nor close enough to prevent circulation of air. The pavilions should be far enough from public roads to be protected from noise and, above all, from dust, for dust often means disease.

It is, I believe, suggested that Ilford will ultimately need a hospital containing 200 beds, and although that number may be at present a long way off, it is the goal which should be kept in view. Such a hospital might certainly be placed on a suitably-shaped plot of four acres, but there would be very little room to spare at some points, and I would therefore venture to urge as strongly as I possibly can that the land purchased be not less than six acres, or, better still, 8 acres. In this, as in so many other things, it is no doubt generally possible to cut our coat according to our cloth; but it does not necessarily follow that the coat will prove to be a complete success when it is made. The best shape for a plot is a parallelogram of about three to one, or, preferably, two to one, having its long axis running east and west. The latter adapts itself well to the usual adjuncts of a hospital, such as the isolation block, the mortuary, the ambulance house, the power house, the laundry, and, if required, the out-patients' department.

The best aspect is one facing south-east, or south, or south-west, the first named being the best of the three, as it permits of the maximum amount of sunshine being obtained. All walls, and especially brick walls, absorb moisture, which is quickly dried out by sunlight, and it is proved that while dampness favours the development of low forms of life, sunshine is a powerful enemy of these spores.

